# Ultra-Thin Multi-Band Polarization-Insensitive Microwave Metamaterial Absorber Based on Multiple-Order Responses Using a Single Resonator Structure

**DOI:** 10.3390/ma10111241

**Published:** 2017-10-27

**Authors:** Yong Zhi Cheng, Zheng Ze Cheng, Xue Song Mao, Rong Zhou Gong

**Affiliations:** 1 School of Information Science and Engineering, Wuhan University of Science and Technology, Wuhan 430081, China; xsmao@wust.edu.cn; 2 School of Electronic and Information Engineering, Hubei University of Science and Technology, Xianning 437100, China; czz8986@126.com; 3 School of Optical and Electronic Information, Huazhong University of Science and Technology, Wuhan 430074, China; rzhgong@hust.edu.cn

**Keywords:** metamaterial absorber, multi-band, polarization-insensitive, circular sector resonator structure

## Abstract

We design an ultra-thin multi-band polarization-insensitive metamaterial absorber (MMA) using a single circular sector resonator (CSR) structure in the microwave region. Simulated results show that the proposed MMA has three distinctive absorption peaks at 3.35 GHz, 8.65 GHz, and 12.44 GHz, with absorbance of 98.8%, 99.7%, and 98.3%, respectively, which agree well with an experiment. Simulated surface current distributions of the unit-cell structure reveal that the triple-band absorption mainly originates from multiple-harmonic magnetic resonance. The proposed triple-band MMA can remain at a high absorption level for all polarization of both transverse-electric (TE) and transverse-magnetic (TM) modes under normal incidence. Moreover, by further optimizing the geometric parameters of the CSRs, four-band and five-band MMAs can also be obtained. Thus, our design will have potential application in detection, sensing, and stealth technology.

## 1. Introduction

Electromagnetic (EM) metamaterials (MMs), as artificial periodic sub-wavelength structured composite materials, have attracted great attention since they can achieve exotic EM or light properties unavailable in nature [1]. Several potential applications, including cloak [2], perfect lens [3], miniaturization devices [4], and so forth have been proposed and demonstrated over a wide EM spectrum range from radio to visible frequency regimes. One of the important applications is to design perfect metamaterial absorbers (MMAs), which can achieve near unity absorption by adjusting the geometric parameters of the MM’s unit-cell structure [5,6]. MMAs are considered to be a branch of MMs, since this has been demonstrated experimentally in the seminal work of Landy et al. that recently provoked extensive interest due to its potential application in thermal emission, solar cells, imaging and sensing [5,6,7,8,9,10,11]. Since then, various MMA structures, such as split-ring and its derivative structure [12,13,14,15], cut-wire [16,17], ring-shape [18,19,20], and cross-shape [21,22,23] have been proposed intensively for the production of near perfect absorption with dual-/triple-/multi-/broad-band in a wide EM-spectrum range. However, there is still a lack of sufficient progress towards the effective design of multi-band and broadband MMAs using a simple polarization-insensitive structure with ultra-thin thickness. 

Some efforts have been made towards the development of multi-band and broadband MMAs by coplanar or stacked multiple-layer structures composed of several different geometrical parameters of the metallic resonant structures [24,25,26,27,28,29,30,31,32,33]. However, these approaches suffer some shortcomings or disadvantages, such as complex resonant structures, larger lattice size and relatively larger thickness, which greatly hamper their practical application. The design strategy of these multi-band and broadband MMAs is mainly based on the combined effect of the fundamental EM resonance of the resonator structures with different parameters. In fact, high-order response of MMs is useful in the design of multi-band MMAs. For example, some multi-band MMAs based on multiple-order magnetic resonance using a single metallic resonant structure have been proposed and investigated [34,35,36,37,38]. However, current designs of MMAs are mainly concentrated in the dual-band and triple-band, most of which are sensitive to the polarization angle of the normal incident EM waves. Thus, it is very important to design a new type of multi-band MMA (where the number of the absorption peaks is more than four) with a simple resonant structure and novel mechanism [39,40]. 

In this work, we firstly present a triple-band polarization-insensitive MMA formed by only a single circular sector resonant (CSR) structure and a ground-plane separated by a dielectric layer based on the multiple-order responses. Simulation results show that the proposed MMA is only 0.8 mm thick and has three absorption peaks over 98% at 3.35 GHz, 8.65 GHz, and 12.44 GHz, respectively, which agrees well with the experiment. The origin of triple-band absorption is clarified by the distributions of the surface current. The triple-band MMA is polarization-insensitive due to the fourfold rotational symmetry of the proposed CSR structure. Then, four-band and five-band MMAs are proposed and demonstrated numerically based on multiple-order responses, by selecting the appropriate geometric parameters of the unit-cell structure.

## 2. Unit-Cell Structure Design, Simulation and Experiment

**Figure 1:**
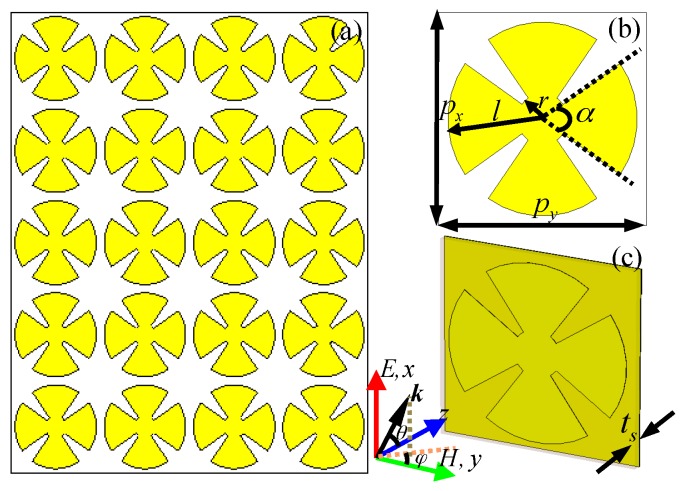
The designed multi-band metamaterial absorber (MMA): (**a**) 2D-array structure; (**b**,**c**) front view and perspective view of the unit cell structure, respectively.

Figure 1 presents the design schematic of the proposed triple-band MMA, which consists of two functional metallic layers separated by a middle spacing layer. As shown in Figure 1b, the front view of the proposed MMA is a single metallic CSR structure as the basic unit cell, which can response the electric field of the incident EM waves. Similar to the previous design [38], the single CSR structure contains a circular patch at the centre and four circular sectors around the circular patch. The middle spacer is the dielectric substrate, and the selections of its thickness and permittivity is crucial since it affects the absorption properties of the MMA. The back metallic layer is a continuous copper film, and its thickness is larger than that of the penetration depth of incident EM waves, thus avoiding transmission. In the design of a triple-band MMA, the FR-4(loss) with permittivity of *ε_r_* = 4.3 × (1 + i0.025) and thickness of 0.8 mm was selected as the middle dielectric substrate, and the continuous copper film with frequency-independent conductivity of *σ* = 5.8 × 10^7^ S/m and thickness of 35 μm were selected as metallic layers. It was expected that the designed MMA is polarization-insensitive for both transverse-electric (TE) and transverse-magnetic (TM) modes due to the fourfold rotational symmetry of its unit-cell structure. The optimized geometric parameters were as follows: *p_x_* = *p_y_* = 20 mm, *t*_s_ = 0.8 mm, *r* = 2.4 mm, *l* = 9 mm, and *α* = 70°.

**Figure 2:**
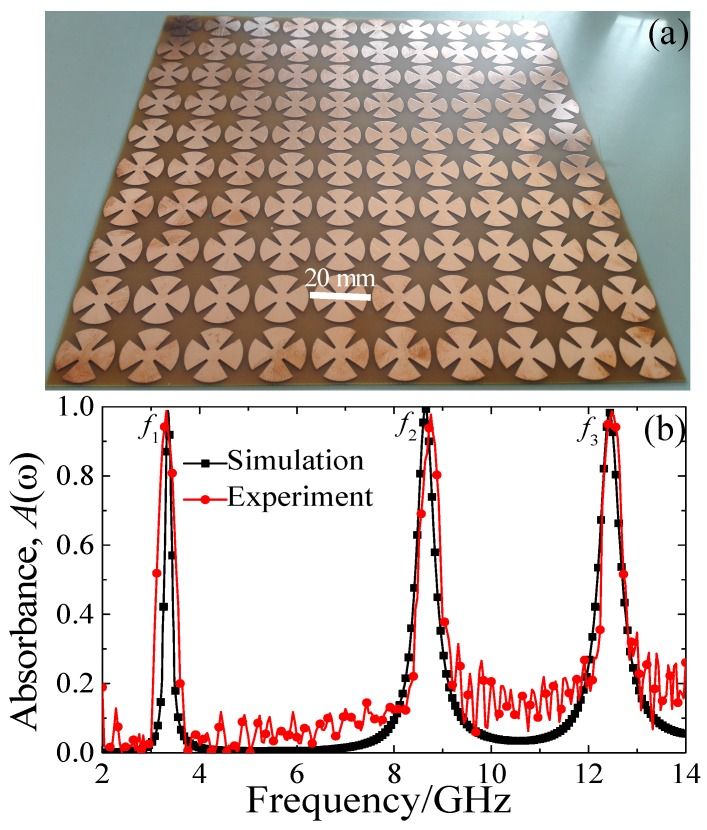
(**a**) Photograph of the fabricated MMA sample; (**b**) the simulated and measured absorbance of the triple-band MMA for normal incidence.

To verify the absorption properties of our design, numerical simulations were performed by using the frequency solver based on the finite integration technology (FIT) in CST Microwave Studio (CST China, LTD., Shanghai, China). In the simulation process, the unit-cell boundary condition was employed along the *x*-axis and *y*-axis direction; the unit-cell structure was illuminated by a normally incident plane wave with the electric field parallel to the *x*-axis and magnetic field parallel to the *y*-axis; and the wave vector was along the +*z* axis direction. For experiments, we fabricated the MMA sample with conventional printed circuit board (PCB) technology. The geometrical parameters and EM parameters of the unit-cell structure of the fabricated MMA were the same as in the simulation. In the MMA sample fabrication, we also used the copper and FR-4(loss) substrate as the metallic structure and dielectric layer, which were also the same as in the simulation. Finally, as shown in Figure 2a, a fabricated MMA sample with an area of 200 mm × 200 mm could be obtained. Two standard horn antennas connected to the network analyzer (Agilent N5244A PNA-X, Agilent Technologies, Santa Clara, CA, USA) were employed to measure the absorbance of the designed MMA in the EM anechoic chamber. In reflection measurement, the source and receiver horn antennas were each inclined at an angle of about 5° with respect to normal on the MMA sample. Before measurement of the sample, the aluminium plate of the same size replaced the sample for reference measurement [12,15]. To eliminate the near-field effect, the distance between the horn antennas and MMA sample was far larger than operational wavelength, which was 2.5 m in the EM anechoic chamber. The absorbance could be calculated through the formula *A* (*ω*) = 1 − *R*(*ω*) − *T*(*ω*) = 1 − |*S*_11_(*ω*)|^2^ − |*S*_21_(*ω*)|^2^, where the *S*_11_(*ω*) and *S*_21_(*ω*) were scattering parameters, and *R*(*ω*) and *T*(*ω*) were reflectance and transmittance as a function of frequency, respectively. In this study, only the reflection was considered, thus the absorbance was calculated only by using *A* (*ω*) = 1 − |*S*_11_(*ω*)|^2^, since the MMA was packed by a continuous film and there was no transmission.

## 3. Results and Discussions

### 3.1. Triple-Band Metamaterial Absorber (MMA)

The simulated and measured absorbance of the proposed MMA in the frequency range of 2–14 GHz is shown Figure 2b. Three resonant absorption peaks were clearly evident: at *f*_1_ = 3.35 GHz, *f*_2_ = 8.65 GHz and *f*_3_ = 12.44 GHz, the simulated absorbance is about 98.8%, 99.7% and 98.3%, respectively, which was in agreement with the experiment. However, there existed slight deviations around the off-resonant frequency range due to the tolerances in fabrication and imperfection in measurements. The simulated Q-factor values of the three absorption peaks were about 19.7, 19.6, and 24.4, respectively. Furthermore, the off-resonance absorbance of the triple-band MMA for both experiment and simulation was small (<30%), which is useful in detecting and sensing applications. The total thickness of the proposed triple-band MMA was only 0.8 mm, which is about λ/119 at 3.35 GHz, λ/43 at 8.65 GHz and λ/30 at 12.44 GHz, respectively, where λ is the corresponding operational wavelength. Thus, our designed triple-band MMA had an ultra-thin property with respect to the operational wavelength.

**Figure 3:**
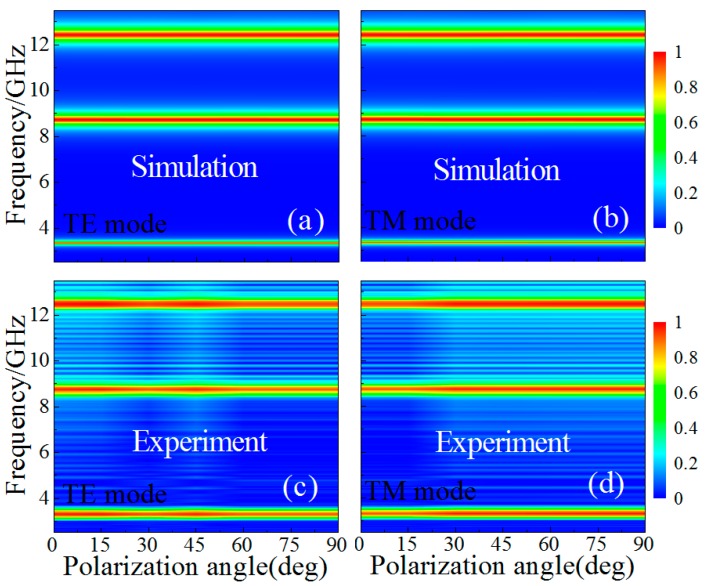
(**a**,**b**) Simulated and (**c**,**d**) experimental absorbance at different polarization angles with different modes: (**a**,**c**) transverse-electric (TE) mode, (**b**,**d**) transverse-magnetic (TM) mode.

We studied, both numerically and experimentally, the polarization angle dependence of the proposed MMA for both TE and TM modes under normal incidence, as shown in Figure 3. From Figure 3a,b, as the polarization angle increased from 0° to 90° in steps of 15° at normal incidence, the simulated absorbance for both the TE and TM modes was unchanged, which was also in good agreement with the experiment (see Figure 3c,d). It was, therefore, further demonstrated that the designed triple-band MMA can keep polarization stability under normal incidence in practical applications. 

**Figure 4:**
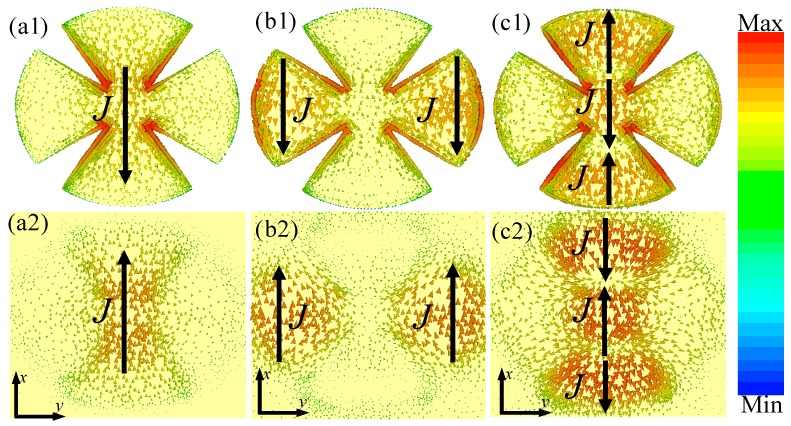
The surface current distributions of the (**a1**–**c1**) front and (**a2**–**c2**) back layer of the unit-cell structure at different resonant frequencies: (**a1**,**a2**) *f*_1_ = 3.35 GHz; (**b1**,**b2**) *f*_2_ = 8.65 GHz; and (**c1**,**c2**) *f*_3_ = 12.44 GHz. The solid arrow indicates the flow directions of surface currents at two metallic layers.

To reveal the physical origin of the proposed triple-band MMA, we studied the surface current distributions of the front and back metallic layer of the unit-cell structure at those resonant absorption frequencies, as shown in Figure 4. It can be seen that the surface current distributions on the front and back metallic layer were in an anti-parallel direction for all three distinct absorption peaks. These anti-parallel currents could form a different circulating current loop, and this was in perpendicular plane to the magnetic field direction of the incident EM waves. Thus, the anti-parallel currents of the front and back metallic layers are illustrations that the absorptions at above the three peaks were induced by magnetic resonance. At the lower frequency (*f*_1_ = 6.68 GHz), as shown in Figure 4(a1,a2), flow directions of surface currents at the front and back metallic layer were opposite, and the antiparallel surface currents could form a current loop between the front and back metallic layers along the magnetic field of the incident EM waves. Thus, the absorption of the lower frequency was induced by the fundamental magnetic response. At the second resonant frequency (*f*_2_ = 8.65 GHz), as shown in Figure 4(b1,b2), the surface currents were composed of two sub-antiparallel currents in the left and right areas of the unit-cell structure, which formed two current loops between the front and back metallic layers. Thus, the second frequency absorption originated from the second-order magnetic resonance mode. At the third resonant frequency (*f*_3_ = 12.44 GHz) from Figure 4(c1,c2), the surface currents composed of three sub-antiparallel currents were at the upper, middle and lower areas of the unit-cell structure, respectively, which formed three current loops between the front and back metallic layers. Obviously, the third absorption was induced by the third-order magnetic resonance mode [17,34,35,36,37]. In effect, the surface current distributions of the unit-cell structure at absorption peak frequencies revealed multipolar responses corresponding to the nature of localized surface plasmon (LSP) behaviors and the excitations of the spoof surface plasmon polaritons (SPPs) [40,41,42]. The spoof SPPs and LSPs were excited by the electric field of the normal incident EM waves, and the incident EM energy was in confinement and concomitant enhancement at metal-dielectric interfaces of the MMAs [40]. Thus, the LSP behaviors and effect of the spoof SPPs could also contribute to the triple-band absorption properties of our proposed structure. Obviously, a novel and simple design of triple-band MMA was easily realized based on the combination of the fundamental and high-order magnetic resonance modes of the unit-cell structure. These results suggest a new approach to designing multi-band MMA by exploring fundamental and high-order magnetic resonance modes in a single patterned resonator structure.

**Figure 5:**
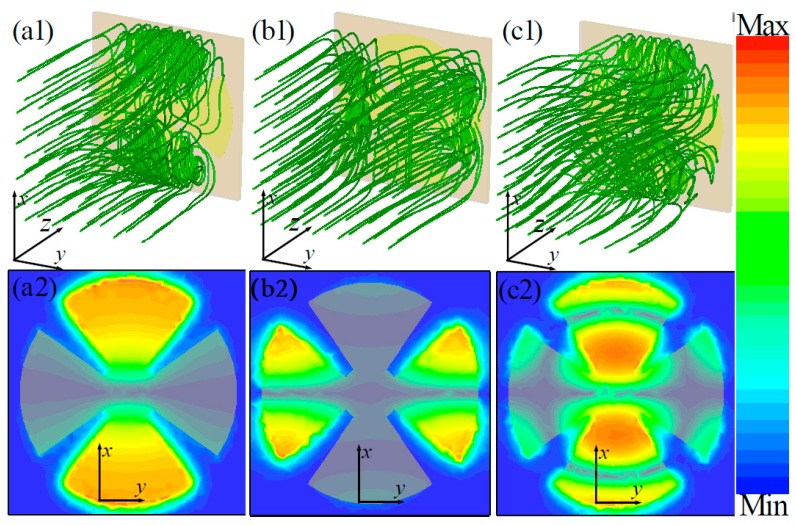
The distributions of (**a1**–**c1**) power-flow streams and (**a2**–**c2**) power-loss density of the unit-cell structure at different resonant frequencies: (**a1**,**a2**) *f*_1_ = 3.35 GHz; (**b1**,**b2**) *f*_2_ = 8.65 GHz; and (**c1**,**c2**) *f*_3_ = 12.44 GHz.

To further illustrate the resonance absorption properties of our proposed triple-band MMA, the distributions of the power-flow streams in the unit cell structure were also studied numerically, since this could provide interesting information about where and how the absorption occurs. From Figure 5(a1–c1), for the three resonant frequencies, the input power-flow streams were originally parallel in the space far from the unit-cell structure in the wave ports. When the power-flow streams moved close to the surface of the unit-cell structure, most of the power-flow streams across the CSR structure curled. However, when the power streams from outside the CSR structure area flowed into the dielectric substrate, this detail was very different at different resonant frequencies. At the lower resonant frequency (*f*_1_ = 3.35 GHz), as shown in Figure 5(a1), the power streams from the outside area into the dielectric substrate mainly focused on the upper and lower edges of the CSR structure. At the second resonant frequency (*f*_2_ = 8.65 GHz), most of the curled power streams were concentered on the left and right edges areas of the structure (see Figure 4(b1)). At the third resonant frequency (*f*_3_ = 12.44 GHz), the curled power streams were mainly concentered on the upper, middle and lower edges areas of the structure (see Figure 4(c1)). In all cases, the power density in the corresponding edge areas was at least one order larger than in other positions of the CSR unit-cell structure.

To better understand the intrinsic absorption properties of the triple-band MMA, the distributions of power-loss density in the middle plane between the two metallic layers of the unit-cell structure at resonant frequencies are given in Figure 5(a2–c2). It can be observed that the power loss was focused on a different area of the middle plane at different resonant frequencies. As shown in Figure 5(a2), at 3.35 GHz, the high power losses were mainly focused on the upper and lower areas of the CSR structure, which was caused by the fundamental magnetic resonance. At the second resonant frequency of 8.65 GHz, as shown in Figure 5(b2), the high power losses were mainly concentered on the left and right areas of the CSR structure originating from the second-order magnetic resonance. At the third resonant frequency of 12.44 GHz, as shown in Figure 5(c2), the high power losses were mainly concentered on the upper and lower areas of the CSR structure due to the third-order resonance mode. 

The distribution properties of power-flow streams and power-loss density are consistent with the surface current distributions of the unit-cell structure. These results further indicate that triple-band perfect absorption is mainly due to the combination of the fundamental and higher-order magnetic resonance responses. In the next section, we extend the analysis of MMAs by making full use of the high-order responses of the CSR structure to achieve multi-band (considering only four-band and five-band MMA, as examples) perfect absorption.

### 3.2. Four-Band and Five-Band MMAs

**Figure 6:**
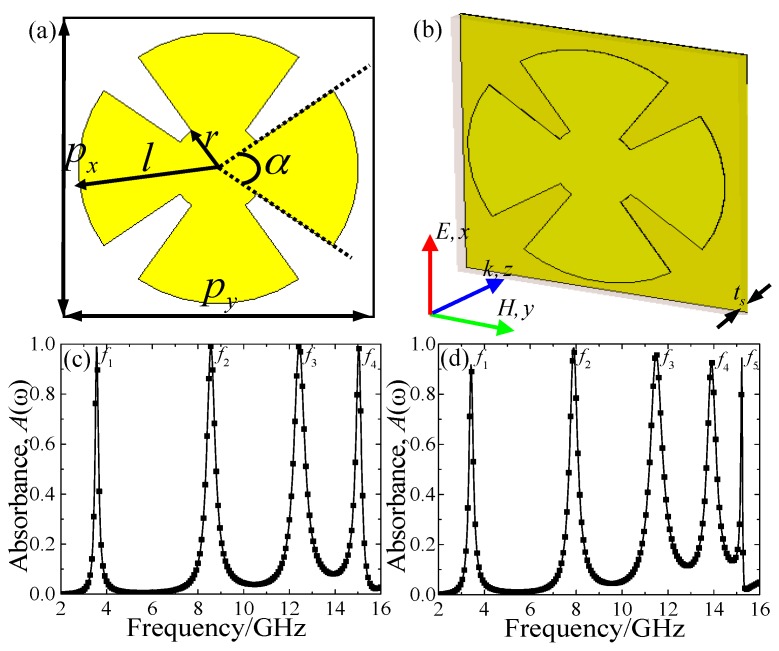
The (**a**) front view and (**b**) perspective view of the unit-cell structure of the designed MMA; with the simulated absorbance for the (**c**) four- and (**d**) five-band MMAs.

The number of perfect absorption peaks of the proposed the MMA structure can be extended based on the combination of one or more high-order responses by selecting appropriate geometric parameters of the unit-cell structure, as shown in Figure 6a,b. In the new designs, the period (*p_x_ = p_y_* = 20 mm), the permittivity of the dielectric substrate, and the conductivity of metallic layers are the same as in Figure 1. For the four-band MMA, the optimized geometric parameters are as follows: *t*_s_ = 1 mm, *r* = 3 mm, *l* = 9.3 mm, and *α* = 70°. For the five-band MMA, the optimized geometric parameters are given as: *t*_s_ = 1.3 mm, *r* = 3.5 mm, *l* = 9.6 mm, and *α* = 75°. The simulated absorbance for the four-band and five-band MMAs are depicted in Figure 6c,d.

**Figure 7:**
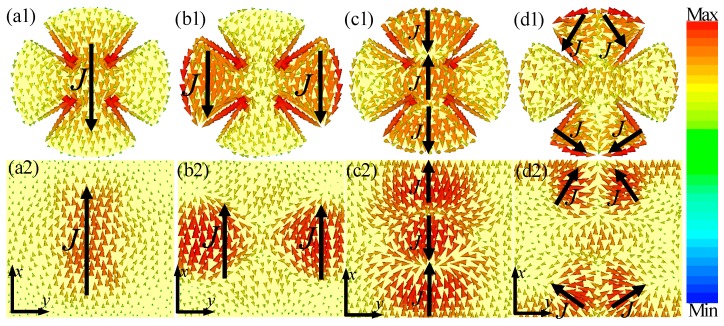
The surface current distributions of the (**a1**–**d1**) front and (**a2**–**d2**) back metallic layers of the unit-cell structure at different resonant frequencies: (**a1**,**a2**) *f*_1_ = 3.56 GHz; (**b1**,**b2**) *f*_2_ = 8.56 GHz; (**c1**,**c2**) *f*_3_ = 12.42 GHz; and (**d1**,**d2**) *f*_4_ = 15.04 GHz. The solid arrow indicates the flow directions of surface currents at the two metallic layers.

**Figure 8:**
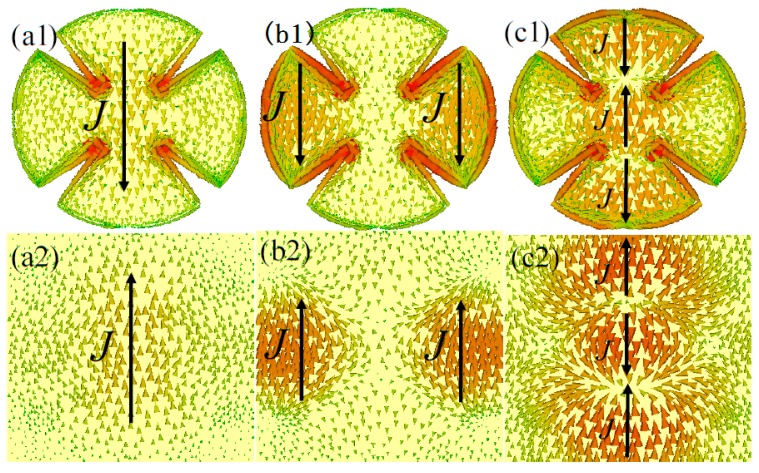

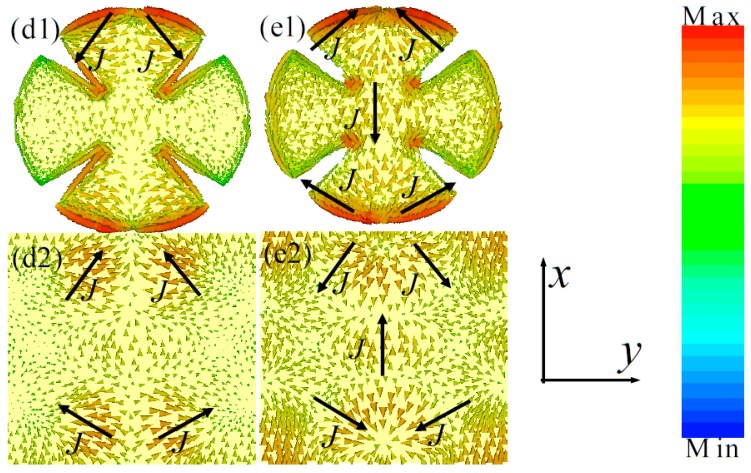
The surface current distributions of the (**a1**–**e1**) front and (**a2**–**e2**) back metallic layer of the unit-cell structure at different resonant frequencies: (**a1**,**a2**) *f*_1_ = 3.42 GHz; (**b1**,**b2**) *f*_2_ = 7.88 GHz; (**c1**,**c2**) *f*_3_ = 11.48 GHz; (**d1**,**d2**) *f*_4_ = 13.9 GHz; and (**e1**,**e2**) *f*_5_ = 15.22 GHz. The solid arrow indicates the flow directions of surface currents at the two metallic layers.

First, we consider the four-band MMA, and the corresponding absorbance is shown in Figure 6c. It can be observed that there were four absorption peaks at discrete resonant frequencies of *f*_1_ = 3.56 GHz, *f*_2_ = 8.56 GHz, *f*_3_ = 12.42 GHz and *f*_4_ = 15.04 GHz; with absorbance of 98.7%, 99.1%, 99.9% and 98.1%, respectively. The corresponding Q values of the four absorption peaks were about 19.8, 19.9, 21.4 and 50.1, respectively. Figure 6d shows the simulated absorbance of the proposed five-band MMA. It can be observed clearly that five absorption peaks were obtained at *f*_1_ = 3.42 GHz, *f*_2_ = 7.88 GHz, *f*_3_ = 11.48 GHz, *f*_4_ = 13.9 GHz, and *f*_5_ = 15.22 GHz; with absorbance of 91.7%, 98.3%, 96.4%, 92.8 and 94.4%, respectively. The corresponding Q values of the five-band MMA were about 16.3, 18.8, 18.5, 33.1 and 253.7, respectively. Furthermore, the absorbance of the four-band and five-band MMAs in the off-resonance frequency were also very small (<20%). Obviously, the proposed four-band and five-band MMAs were also polarization-insensitive due to the fourfold rotational symmetry. In addition, we can conjecture that the near perfect absorption peaks were mainly attributed to the overlapping of the four- and five-order magnetic resonance responses for the four-band and five-band MMA structures (see Figure 7 and Figure 8).

To better understand the physical origin of the observed four-band and five-band absorption properties, the surface current distributions of the front and back metallic layer of the unit-cell structure corresponding to the absorption peaks are given in Figure 7 and Figure 8. As shown in Figure 7(a1–c1,a2–c2), it is obvious that the surface current distributions of the front and back metallic layers of the unit-cell structure were nearly the same as those in Figure 4. Thus, at *f*_1_ = 3.56 GHz, *f*_2_ = 8.56 GHz and *f*_3_ = 12.42 GHz, near perfect absorption was caused by the fundamental, second-order and third-order magnetic responses of the proposed MMA structure. Furthermore, at the fourth resonant frequency (*f*_4_ = 15.04 GHz), as shown in Figure 7(d1,d2), four sub-antiparallel currents of the unit-cell structure could form four current loops between the front and back metallic layers that gave rise to the excitation of four magnetic resonances in the dielectric layer of the MMAs. Thus, the fourth absorption was induced by the fourth-order magnetic resonance mode. Therefore, quad-band perfect MMA could be obtained based on the combination of the fundamental, second-order, third-order, and fourth-order resonance of the proposed CSR structure.

Obviously, it is possible that the five absorption peaks were induced by the combination of the fundamental, second-order, third-order, fourth-order, and fifth-order magnetic resonance responses of the designed four-band MMA structure.

As shown in Figure 8(a1–d1,a2–d2), the distribution properties of the surface current of the front and back metallic layers of the unit-cell structure are similar to those in Figure 7. Thus, at resonant frequencies of *f*_1_ = 3.42 GHz, *f*_2_ = 7.88 GHz, *f*_3_ = 11.48 GHz, and *f*_4_ = 13.9 GHz, the high-level absorption properties mainly originated from the fundamental, second-order, third-order and fourth-order magnetic response of the proposed MMA structure. In addition, as shown in Figure 8(e1,e2) for the fifth resonant frequency (*f*_5_ = 15.22 GHz), five sub-antiparallel currents at the unit-cell structure form five current loops between the front and back metallic layers, resulting in the fifth-order magnetic resonance mode. Thus, five absorption peaks can be achieved easily based on the combination of the fundamental and higher-order magnetic resonance responses of the proposed MMA structure. Therefore, we also suggest that the LSP behaviors and effect of the spoof SPPs contribute to the four-and five-band absorption properties of our proposed MMA structure.

These results suggest that the combination of fundamental and higher-order magnetic resonances in a simple resonant structure can lead to the superposition of their absorption spectrum, confirming the idea of creating multi-band MMAs. The presented four-band and five-band MMAs are also easy to fabricate using conventional PCB technology. Furthermore, we can conjecture that the numbers of absorption peaks (i.e., six-order and seven-order resonances) of the proposed MMA structure can be further extended by selecting the appropriate geometric parameters of the unit-cell structure. Thus, it is expected that a multi-band (more than five) MMA can also be achieved based on the combination of one or more high-order resonances. However, it should be noted that the absorbance of the MMA will be smaller (<90%) to some degree when the number of absorption peaks is more than five (not shown).

## 4. Conclusions

In conclusion, the absorption properties of multi-band MMAs using a single circular sector resonator structure based on the combination of fundamental and higher-order magnetic resonance were studied. Firstly, a detailed example of a triple-band perfect MMA that was ultra-thin and polarization-insensitive was demonstrated by simulation and experiment. Simulations confirmed that the absorbance of the MMA was more than 99% on average at three different resonant frequencies, which agreed well with the experiment. The total thickness of the proposed triple-band MMA was only 0.8 mm, which was smaller than λ/30 at 12.44 GHz, where λ is the corresponding operational wavelength. The absorption of proposed triple-band MMA was unchanged for all polarization angles of both TE and TM waves. The simulated surface current distributions indicated that triple-band perfect absorption mainly originated from the fundamental, second-order, and third-order magnetic resonances.

Furthermore, we could achieve four-band and five-band near perfect absorption at the desired frequency ranges by selecting the appropriate geometric parameters of the proposed MMA unit-cell structure, thus extending our idea to four-band and five-band MMAs. Moreover, we can conjecture that the proposed CSR structure can be used to further extend the number of absorption peaks (i.e., six-order and seven-order resonances) by combining one or more high-order resonances. In addition, the multi-band MMA that is polarization-insensitive and ultra-thin can be realized easily in terahertz, infrared and even visible frequency regions due to its geometry scalability. It is also expected that our work might contribute a simple and effective method for creating novel and high-performance multiband MMAs. Thus, our simple design of a multi-band MMA may find some potential applications in spatial filters, wavelength selective radiation, sensing, detecting, and stealth technology. 

## References

[1] Cui T.J., David R.S., Liu R. (2010). Metamaterials: Theory, Design, and Applications.

[2] Liu R., Ji C., Mock J.J., Chin J.Y., Cui T.J., Smith D.R. (2009). Broadband Ground-Plane Cloak. Science.

[3] Xu T., Agrawal A., Abashin M., Chau K.J., Lezec H.J. (2013). All-angle negative refraction and active flat lensing of ultraviolet light. Nature.

[4] Rahimi M., Zarrabi F.B., Ahmadian R., Mansouri Z., Keshtkar A. (2014). Miniaturization of antenna for wireless application with difference metamaterial structures. Prog. Electromagn. Res..

[5] Landy N.I., Sajuyigbe S., Mock J.J., Smith D.R., Padilla W.J. (2008). Perfect metamaterial absorber. Phys. Rev. Lett..

[6] Watts C.M., Liu X., Padilla W.J. (2012). Metamaterial electromagnetic wave absorbers. Adv. Mater..

[7] Pu M.B., Chen P., Wang Y.Q., Zhao Z.Y., Wang C.T., Huang C., Hu C.G., Luo X.G. (2013). Strong enhancement of light absorption and highly directive thermal emission in graphene. Opt. Express.

[8] Tang C.J., Yan Z.D., Wang Q.G., Chen J., Zhu M.W., Liu B., Liu F.X., Sui C.H. (2015). Ultrathin amorphous silicon thin-film solar cells by magnetic plasmonic metamaterial absorbers. RSC Adv..

[9] Carranza I.E., Grant J.P., Gough J., Cumming D. (2017). Terahertz metamaterial absorbers implemented in CMOS technology for imaging applications: Scaling to large format focal plane Arrays. IEEE J. Sel. Top. Quantum Electron..

[10] Cheng Y.Z., Mao X.S., Wu C.J., Wu L., Gong R.Z. (2016). Infrared non-planar plasmonic perfect absorber for enhanced sensitive refractive index sensing. Opt. Mater..

[11] Dayal G., Chin X.Y., Soci C., Singh R. (2017). High-Q plasmonic fano resonance for multiband surface-enhanced infrared absorption of molecular vibrational sensing. Adv. Opt. Mater..

[12] Cheng Y.Z., Yang H.L., Cheng Z.Z., Wu N. (2011). Perfect metamaterial absorber based on a split-ring-cross resonator. Appl. Phys. A.

[13] Xu Y.Q., Zhou P.H., Zhang H.B., Chen L., Deng L.J. (2011). A wide-angle planar metamaterial absorber based on split ring resonator coupling. J. Appl. Phys..

[14] Li S., Gao J., Cao X., Zhang Z., Zheng Y., Zhang C. (2015). Multiband and broadband polarization-insensitive perfect absorber devices based on a tunable and thin double split-ring metamaterial. Opt. Express.

[15] Zhao J.C., Cheng Y.Z. (2016). Ultrabroadband microwave metamaterial absorber based on electric SRR loaded lumped resistors. J. Electron. Mater..

[16] Hu C.G., Li X., Feng Q., Chen X.N., Luo X.G. (2010). Investigation on the role of the dielectric loss in metamaterial absorber. Opt. Express.

[17] Yoo Y.J., Kim Y.J., Hwang J.S., Rhee J.Y., Kim K.W. (2015). Triple-band perfect metamaterial absorption based on single cut-wire bar. Appl. Phys. Lett..

[18] Shen X., Cui T.J., Zhao J., Ma H.F., Jiang W.X., Li H. (2011). Polarization-independent wide-angle triple-band metamaterial absorber. Opt. Express.

[19] Park J.W., Tuong P.V., Rhee J.Y., Kim K.W. (2013). Multi-band metamaterial absorber based on the arrangement of donut-type resonators. Opt. Express.

[20] Wang B.X., Zhai X., Wang G.Z., Huang W.Q., Wang L.L. (2015). Design of a four-band and polarization-insensitive terahertz metamaterial absorber. IEEE Photonics J..

[21] Liu X., Starr T., Starr A.F., Padilla W.J. (2010). Infrared spatial and frequency selective metamaterial with near-unity absorbance. Phys. Rev. Lett..

[22] Cheng Y.Z., Nie Y., Gong R.Z. (2013). Metamaterial absorber and extending absorbance bandwidth based on multi-cross resonators. Appl. Phys. B.

[23] Ma W., Wen Y., Yu X. (2013). Broadband metamaterial absorber at mid-infrared using multiplexed cross resonators. Opt. Express.

[24] Shen X.P., Yang Y., Zang Y., Gu J., Han J., Zhang W., Cui T.J. (2012). Triple-band terahertz metamaterial absorber: Design, experiment, and physical interpretation. Appl. Phys. Lett..

[25] Li H., Yuan L.H., Zhou B., Shen X.P., Cheng Q., Cui T.J. (2011). Ultrathin multiband gigahertz metamaterial absorbers. J. Appl. Phys..

[26] Cheng Y.Z., Nie Y., Gong R.Z., Yang H.L. (2011). Multi-band metamaterial absorber using cave-cross resonator. Eur. Phys. J. Appl. Phys..

[27] Cheng Y.Z., Nie Y., Gong R.Z. (2013). A polarization-insensitive and omnidirectional broadband terahertz metamaterial absorber based on coplanar multi-squares films. Opt. Laser Technol..

[28] Kollatou T.M., Dimitriadis A.I., Assimonis S.D., Kantartzis N.V., Antonopoulos C.S. (2014). Multi-band highly absorbing microwave metamaterial structures. Appl. Phys. A.

[29] Ye Y.Q., Jin Y., He S.L. (2010). Omnidirectional, polarization-insensitive and broadband thin absorber in the terahertz regime. J. Opt. Soc. Am. B.

[30] Ding F., Cui Y.X., Ge X.C., Zhang F., Jin Y., He S.L. (2012). Ultra-broadband microwave metamaterial absorber. Appl. Phy. Lett..

[31] Liang Q.Q., Yu W.X., Zhao W., Wang T., Zhao J., Zhang H., Tao S. (2013). Numerical study of the meta-nanopyramid array as efficient solar energy absorber. Opt. Express.

[32] Zheng D.H., Cheng Y.Z., Cheng D.F., Nie Y., Gong R.Z. (2013). Four-band polarization-insensitive metamaterial absorber based on flower-shaped structures. Prog. Electromagn. Res..

[33] Yin X., Chen L., Li X. (2015). Ultra-broadband super light absorber based on multi-sized tapered hyperbolic metamaterial waveguide arrays. J. Lightw. Technol..

[34] Yoo Y.J., Kim Y.J., Pham V.T., Rhee J.Y., Kim K.W., Jang W.H., Kim Y.H., Cheong H., Lee Y.P. (2013). Polarization-independent dual-band perfect absorber utilizing multiple magnetic resonances. Opt. Express.

[35] Kim Y.J., Yoo Y.J., Kim K.W., Rhee J.Y., Kim Y.H., Lee Y.P. (2015). Dual broadband metamaterial absorber. Opt. Express.

[36] Dung N.V., Tuong P.V., Yoo Y.J., Kim Y.J., Tung B.S., Lam V.D., Rhee J.Y., Kim K.W., Kim Y.H., Chen L.Y. (2015). Perfect and broad absorption by the active control of electric resonance in metamaterial. J. Opt..

[37] Kim S.J., Yoo Y.J., Kim Y.J., Lee Y.P. (2017). Triple-band metamaterial absorption utilizing single rectangular hole. Opt. Commun..

[38] Lee D.J., Hwang J.G., Lim D., Hara T., Lim S. (2016). Incident angle-and polarization-insensitive metamaterial absorber using circular sectors. Sci. Rep..

[39] Govind D., Ramakrishna S.A. (2014). Multipolar localized resonances for multi-band metamaterial perfect absorbers. J. Opt..

[40] Cheng Y.Z., Huang M.L., Chen H.R., Guo Z.Z., Gong R.Z., Mao X.S. (2017). Ultrathin six-band polarization-insensitive perfect metamaterial absorber based on a cross-cave patch resonator for terahertz waves. Materials.

[41] Liao Z., Luo Y., Fernández-Domínguez A.I., Shen X.P., Maier S.A., Cui T.J. (2015). High-order localized spoof surface plasmon resonances and experimental verifications. Sci. Rep..

[42] Park M.S., Bhattarai K., Kim D.K., Kang S.W., Kim J.O., Zhou J.F., Jang W.Y., Noyola M., Urbas A., Ku Z. (2014). Enhanced transmission due to antireflection coating layer at surface plasmon resonance wavelengths. Opt. Express.

